# Influence of MoS_2_ Nanosheet Size on Performance of Drilling Mud

**DOI:** 10.3390/polym11020321

**Published:** 2019-02-13

**Authors:** Sung Hyun Hong, Hae Jin Jo, Min-Ju Choi, Ho Won Jang, Young Ju Kim, Wook Ryol Hwang, Soo Young Kim

**Affiliations:** 1 School of Chemical Engineering and Materials Science, Chung-Ang University, 84 Heukseok-ro, Dongjak-gu, Seoul 06974, Korea; hongthomas91@cau.ac.kr; 2 School of Mechanical Engineering, Research Center for Aircraft Parts Technology (ReCAPT), Gyeongsang National University, Jinju 52828, Korea; haejinjo@gnu.ac.kr; 3 Research Institute of Advanced Materials, Department of Materials Science and Engineering, Seoul National University, Seoul 08826, Korea; choiminju@snu.ac.kr (M.-J.C.); hwjang@snu.ac.kr (H.W.J.); 4 Korea Institute of Geoscience and Mineral Resources, 905 Yeongilman-daero, Heunghae-eup, Buk-gu, Pohang-si, Gyeongsangbuk-do 37559, Korea

**Keywords:** water-based drilling mud, 2D material, thermal conductivity, shear thinning

## Abstract

Water-based drilling mud (WBM) is a non-Newtonian fluid that has a variety of applications such as in transporting cuttings during drilling, protecting the borehole, and cooling the drill bit. With the development of nano-technology, various nanoparticles have been synthesized and have been added to WBM to improve its performance. Shear thinning is the most important factor in drilling mud and this attribute can be improved when two-dimensional particles are added. MoS_2_ nanoparticles, which represent a typical two-dimensional material, are easy to synthesize in large quantities and have a high thermal conductivity and low coefficient of friction. Since the two-dimensional structure, thermal conductivity, and low coefficient of friction of MoS_2_ would improve the performance of WBM, we experimented with MoS_2_ nanosheets as an additive, under optimal conditions, using various samples each with uniform sizes and thicknesses of nanosheets. A large amount of MoS_2_ nanosheets was synthesized, sorted by thickness and diameter, and added to drilling mud. The diameter of MoS_2_ was divided into a small diameter group (about 100–400 nm) and a big diameter group (about 300–650 nm), and the thickness was divided into 1–2 nm and 5–10 nm groups. Experimental results showed that when MoS_2_ is added to WBM, shear thinning occurs more strongly. In addition, the addition of MoS_2_ with a thickness of 1–2 nm and a diameter of 300–650 nm resulted in the highest increase in viscosity and thermal conductivity of WBM. As a result, we experimentally confirmed that MoS_2_ can be used as an additive to increase the thermal conductivity and viscosity of WBM and to make shear thinning phenomenon more.

## 1. Introduction

**Figure 1:**
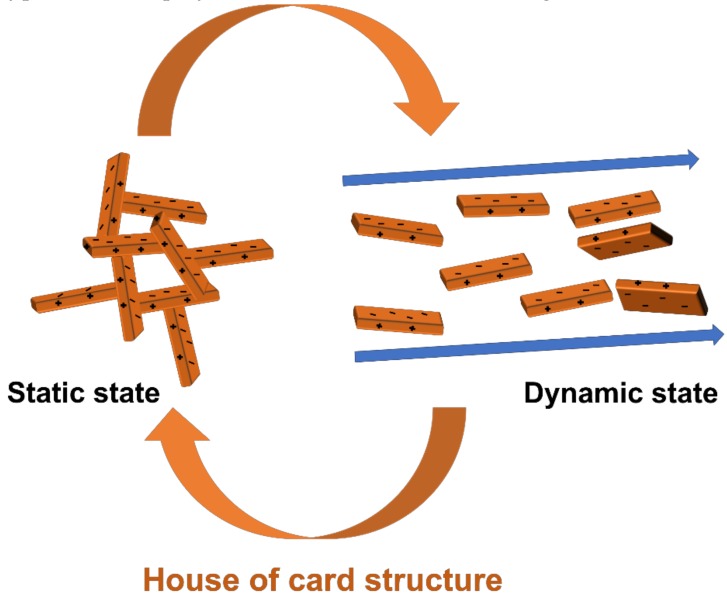
Image about when bentonite is in a static state and a dynamic state.

The drilling fluid commonly referred to as drilling mud is used in drilling operations to remove cuttings, control the pressure of the borehole, lubricate and cool the bit and the drill string, form mud cake, and prevent fluid loss [1]. There are various types of drilling muds, such as water-based mud (WBM), oil-based mud (OBM), synthetic-based mud (SBM), and low-density drilling fluid. OBM and SBM exhibit good performance, however, they are more costly than WBM and can cause environmental problems, while low-density drilling fluid is used only in special cases [2]. WBM is the most common mud and is used in many drilling applications. WBM usually consists of bentonite, barite, and polymer added to fresh or saline water. Bentonite shows a bipolar property where the edges of each layer are positive and the surface is negative. For this reason, when bentonite is present in water, each edge part and surface part are partially connected to each other by electrical properties to make a specific structure. This structure is called the house of cards structure [3]. The fluid containing bentonite has a viscosity increase due to this structure under static conditions, and when the flow rate of the fluid is fast, the structure is disturbed and the viscosity drops sharply. This process which the viscosity is high when the flow rate is low and the viscosity is low when the flow rate is high occurs as a reversible process. One of the reasons for the shear thinning phenomenon is that of the house of cards structure. (Figure 1) Barite increases the density of WBM, while typical viscous polymer additives, such as xanthan gum, increase its viscosity [4,5,6].

WBM needs to possess various properties, depending on the drilling application in question. Typically, WBM performance needs to be checked during the drilling operation. In case of density, it is necessary that the WBM facilitates maintenance of borehole pressure, while in order to transfer cuttings after penetration, it needs to retain a suitable viscosity. If the viscosity is low, the pressure loss is small, and it is easy to circulate the WBM, however cuttings transport remains an issue, due to low viscosity. High WBM viscosity affects the flow rate, and the all-important shear thinning behavior requires appropriate viscosity. Shear thinning behavior is the most important property of drilling fluid, because when the flow velocity of WBM is low, the viscosity is high and the cuttings are transferred smoothly, and, in contrast, when the flow velocity is high, viscosity is low, which increases the flow rate of WBM, and drilling proceeds well [3,4,5]. In addition, WBM must be an effective lubricant to reduce damage to drill bits, while high thermal conductivity is required, to prevent drill bit overheating [7,8]. In addition, WBM needs to facilitate production of a filter cake layer, to prevent fluid loss [9].

Developments in nanotechnology have led to reports that adding various nanoparticles improved WBM performance. Hydrophilic nanoparticles, such as Fe_2_O_3_ or SiO_2_, have a high surface area per unit mass, resulting in increased viscosity and shear stress of the fluid. Reports have shown that the smaller the nanoparticle, the greater the increased viscosity and shear stress [10,11], while in other cases, nanoparticles were added to WBM to increase shear thinning behavior. Especially shear thinning behaviors of drilling mud when TiO_2_ is added are shown linearly with increasing TiO_2_ addition [12,13,14,15,16]. In drilling operations, fluid loss caused by penetration of drilling mud into the reservoir gap has been reduced by adding nanoparticles; in one case, the necessary thickness of the filter cake was reduced by adding two-dimensional, Graphene oxide (GO) particles, which led to reduced fluid loss through raised filter cake impermeability. GO has excellent mechanical strength and good dispersion in water and is also stable. In addition, drilling mud has often alkalinity and GO can be well dispersed in alkalinity so it is used as a drilling mud additive [9,17,18,19]. The properties of WBM can be improved by selecting for specific nanoparticle properties, and it has been demonstrated, for example, that the thermal conductivity of WBM was improved by adding nanoparticles with high thermal conductivity [8,16]. In the case of Copper oxide (CuO) not only increases the thermal conductivity of drilling mud but also has stability in high temperature and high-pressure condition (HPHT) [16]. In addition, nano-clay such as palygorskite (Pal) showed a noticeable increase in viscosity of drilling mud even when added in small amounts, and was stable also in HPHT condition [13].

Transition metal chalcogenides (TMD) are substances in which groups 4 to 10 transition metals, and group 16 gases, are bonded. MoS_2_ is a representative TMD material, with a two-dimensional structure, and a van der Waals bond between layers; it also exhibits an extremely low coefficient of friction, and it is used as an additive in lubricating solutions [20]. Previous research showed that MoS_2_, when added to OBM and WBM, improved the lubricant performance of the drilling mud [21,22].

The electrical and thermal properties of MoS_2_ depend on the phase, size, and thickness of its structure. The theoretical thermal conductivities of 1T phase, single layer, and 2H phase, single layers of MoS_2_, are 32 ± 3 W/m·K, and 40 ± 4 W/m·K, respectively [23]. There has also been a theoretical result in relation to differences in the thermal conductivity of 2H phase MoS_2_, depending on whether it was present as a single layer or the bilayer, of 34.5 W/m·K, and 54 W/m·K, respectively. Although the theoretical thermal conductivity of MoS_2_ has differed between reports, results have consistently shown that the thicker the layer and the larger the particle diameter, the higher the thermal conductivity. It has been experimentally demonstrated that thermal conductivity is 85–110 W/m·K, for bulk, 2H MoS_2_ [24,25,26,27,28,29,30]. The thickness of MoS_2_ also affects the coefficient of friction, with thicker MoS_2_ layers reportedly giving lower coefficients of friction [31].

**Figure 2:**
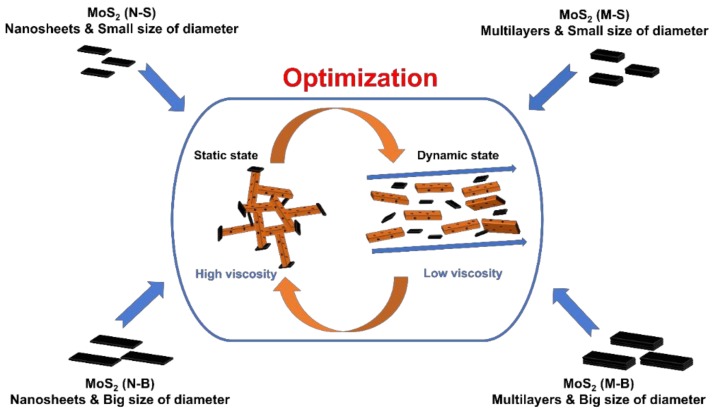
WBM synthesized by adding MoS_2_ with different size and thickness to the base fluid.

In previous studies, MoS_2_ was added to WBM to make a lubricant, but the addition of a small amount (<1 wt %) did not affect viscosity or shear stress. We added bulk MoS_2_ to increase WBM thermal conductivity_._ The interaction between bentonite and MoS_2_ makes high viscosity of the WBM when the WBM is in a static state, and low friction coefficient of MoS_2_ cause viscosity of WBM to drop sharply in a dynamic state (Figure 2). We synthesized MoS_2_ nanoparticles, using two representative methods: ultrasonication and Li-intercalation [32,33,34]. The MoS_2_ nanosheets synthesized by the Li-intercalation method were divided into small and large diameter sheets (‘Nanosheets-Small’ and ‘Nanosheets-Big’: N-S and N-B, respectively). For MoS_2_ multilayered sheets obtained through sonication, we divided the products into small and large diameter, multilayer sheets (‘Multilayers-Small’, and ‘Multilayers-Big’: M-S and M-B respectively). The four synthesized sheet samples (N-S, N-B, M-S, and M-B) were added to the base fluid, as shown in Figure 2. 

If the same concentration of the different MoS_2_ samples was added, the surface area per unit mass of MoS_2_ would be different, due to the difference in size, as the addition of MoS_2_ nanosheets will give rise to a larger surface area than the addition of MoS_2_ multilayers. However, the thicker the MoS_2_, the higher the thermal conductivity and the lower the coefficient of friction, and we tried to resolve performance variations brought about by differences between surface areas through experimentation. 

The base fluids, consisting of water, bentonite, and various amounts of MoS_2_ nanoparticles, are shown in Table S1, together with their measured rheological properties and thermal conductivity. We experimented which thickness and diameter of the MoS_2_ additions, in terms of their ability to enhance the thermal conductivity and rheological properties for WBM, and generally investigated whether MoS_2_ could be a multifunctional nano-additive, capable of bringing other improvements to WBM. 

## 2. Experimental Procedures

### 2.1. Preparation of Base Fluid and MoS_2_ Nanoparticles

The base fluid consisted of 5 wt % bentonite and deionized water (DI water). Bentonite was supplied by Clariant Korea (Seoul, Korea). FE-SEM image of bentonite is attached to Supplementary Materials (Figure S1). The reason for using only bentonite and DI water as base fluid is to confirm the rheological properties of MoS_2_. MoS_2_ was prepared in four different sizes (N-S, N-B, M-S, and M-B) and added to the base fluid at different concentrations (1 wt %, 3 wt %, and 5 wt %). The MoS_2_ Nanosheet was fabricated by the Li-intercalation method and then the diameter was controlled by adjusting the centrifugal speed and the sonication power. MoS_2_ Multilayers adjusted the size of the particles by applying only sonication power to the MoS_2_ raw material. Information on the components in WBM, with MoS_2_ nanoparticles, can be found in Table S1.

### 2.2. Synthesizing MoS_2_ Nanosheets and Size Control

MoS_2_, Hexane, and Butyl lithium were purchased from Sigma-Aldrich. In this work, we used the Li-intercalation method to prepare the MoS_2_ nanosheet published previously [34]. Ten gram MoS_2_ (<2 µm) was added to 33 mL of n-Hexane and 17 mL of n-Butyllithium solution (2.5 M in hexanes), and stored in a container filled with nitrogen gas, for 120 h. It was then centrifuged at 8000 RPM for 2 min, after which the precipitate was retained and the suspension was discarded. This process was repeated twice, and the precipitate was dried on a hotplate until the hexane evaporated. DI water was then added, the solution was sonicated for 1 h in a sonic bath, and centrifuged for 5 min at 8000 RPM, after which the precipitate was retained and the suspension was discarded. This was again repeated twice, adding DI water, centrifuging, and discarding the liquid. Subsequently, the MoS_2_ precipitated by centrifugation, at 500 RPM for 5 min, was referred to as a large-sized MoS_2_ nanosheet (N-B). For the next group, after centrifugation at 8000 RPM for 5 min, the precipitate was sonicated at 60 W for 1 h by ultrasonicator (SONICS VCX0750, Sonics & Materials, Newtown, CT, USA), and was then classified as small-sized MoS_2_ nanosheets (N-S). After creation, the MoS_2_ nanoparticles were re-dispersed in a sonic bath, before adding to the base fluid.

### 2.3. Synthesizing MoS_2_ Multilayers and Size Control

The sonication method is a method of exfoliation MoS_2_ using ultrasonic waves. The probe-ultrasonicator was used for that method. Each aliquot of the required MoS_2_ was placed in a 70 mL vial, with DI water, and was then sonicated, for 1 h at 60 W, or 30 min at 10 W. Each was classified as either multilayered, small-sized MoS_2_ (M-S), or multilayered, big-sized MoS_2_ (M-B). The MoS_2_ nanoparticles were then added directly to the base fluid.

### 2.4. Characterization

Field-emission scanning electron microscopy (FE-SEM, SIGMA/ Carl Zeiss 300 VP, Carl Zeiss, Oberkochen, Germany) was used to measure the structures and sizes of the created MoS_2_ nanoparticles, and UV-vis absorption spectra (V-670 UV-Vis spectrophotometer, JASCO, Mary’s Court Eaaston, MD, USA) were measured to analyze their optical properties. Size distributions of the MoS_2_ nanoparticles were measured by Dynamic Light Scattering (Otsuka ELSZ-1000, Otsuka electronics, Osaka, Japan), and their thicknesses were determined using contact-mode atomic force microscopy (AFM, XE-100, Park systems, Suwon, Korea). For the fluid containing nanoparticles, the transition hot wire method was used to measure thermal conductivity, with a KD2 pro (Decagon, Pullman, WA, USA) used for the actual thermal conductivity measurements. Transition hot wire method is the most common way to measure nanofluid’s thermal conductivity. When the probe gets electric power, the temperature of the wire rises. The method of calculating the thermal conductivity using the temperature gradient characteristic of this wire is called the transition hot wire method [35,36,37,38]. The thermal conductivity is calculated as follows.

(1) $$\lambda \;=\;\frac{q\cdot \ln\frac{t_{2}}{t_{1}}}{4\pi \;\left(\;T_{2}-T_{1\;}\right)}$$

*λ* = Thermal conductivity of the sample (W/m·K), *q* = Generated heat per unit length of sample/time (W/m), *t*_1_,*t*_2_ = measure time length (sec), *T*_1_, *T*_2_ = Temperature at *t*_1_, *t*_2_ (K).

Thermal conductivity was measured three times, and the average value was rounded off to the fourth digit. Rheological property is a term which generally refers to changes in viscosity and yield stress, with shear rate. In this study, a rheometer (Anton Paar MCR 301, Anton Paar Graz, Austria) was used to measure the rheological properties of drilling mud, and PP 50 (parallel plate with diameter 50 mm) was used for the measurement value. A parallel plate accessory (PP 50) was characterized by the flat parts touching the fluid, and it can be confirmed that the samples were stable when measured. By using PP 50, the rheological properties of the various drilling mud compounds tested were measured by using strain control. Shear rate control is an experimental method to measure shear stress with varying shear rate. In this study, shear stress was measured in a shear rate range of between 0.008/s to 1000/s. The viscosity was then calculated by dividing the measured stress by the applied shear rate.

## 3. Results and Discussion

### 3.1. Synthesis and Characterization of MoS_2_

**Figure 3:**
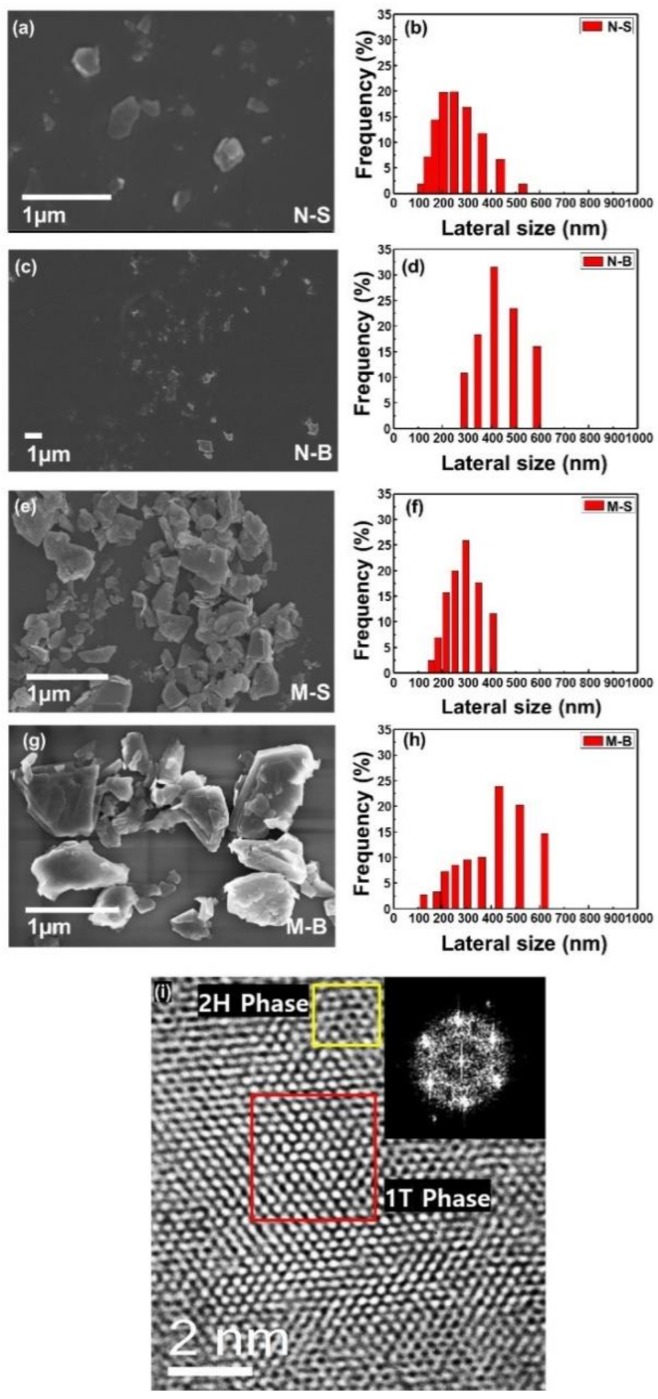
FE-SEM images and DLS graphs of MoS_2_ N-S (**a**,**b**), N-B (**c**,**d**), M-S (**e**,**f**), and M-B (**g**,**h**). The scale bar indicates 1 µm in all images of FE-SEM. In order to compare the diameter distribution of MoS_2_, the lateral size and frequency scale were made equal. TEM and electron diffraction images indicate the phase of MoS_2_ (**i**).

Figure 3 shows FE-SEM images and DLS graphs of the fabricated MoS_2_ nanoparticles. The particle size of the MoS_2_ nanosheets was divided by sonication power and centrifuge speed. When the MoS_2_ nanosheets were precipitated at 500 rpm, the average size distribution of N-B was found to be between 300 and 600 nm, with up to 1 µm size confirmed, by FE-SEM. After the MoS_2_ was exfoliated, no strong sonication was performed, so that the MoS_2_ flakes were large, and only the heavier nanoparticles submerged enough to precipitate at low centrifuge speed. In contrast, when N-S was precipitated, at the relatively high speed of 8000 rpm, and ultrasonicated, particles diameters over 100 nm were found, with the majority within the range of 100–400 nm. The diameter distributions of the MoS_2_ multilayers were also distinguished by the different ultrasonication power and times applied: M-S had 100–400 nm particles, while M-B had a size range of 400–650 nm, while using FE-SEM revealed particles greater than 1 µm. Creating the different sized MoS_2_ was found to be relatively simple, by varying the ultrasonication power. TEM was also measured and the data showed 1T and 2H phases on MoS_2_ surface. The hexagonal structure of MoS_2_ was confirmed by an electron diffraction image.

**Figure 4:**
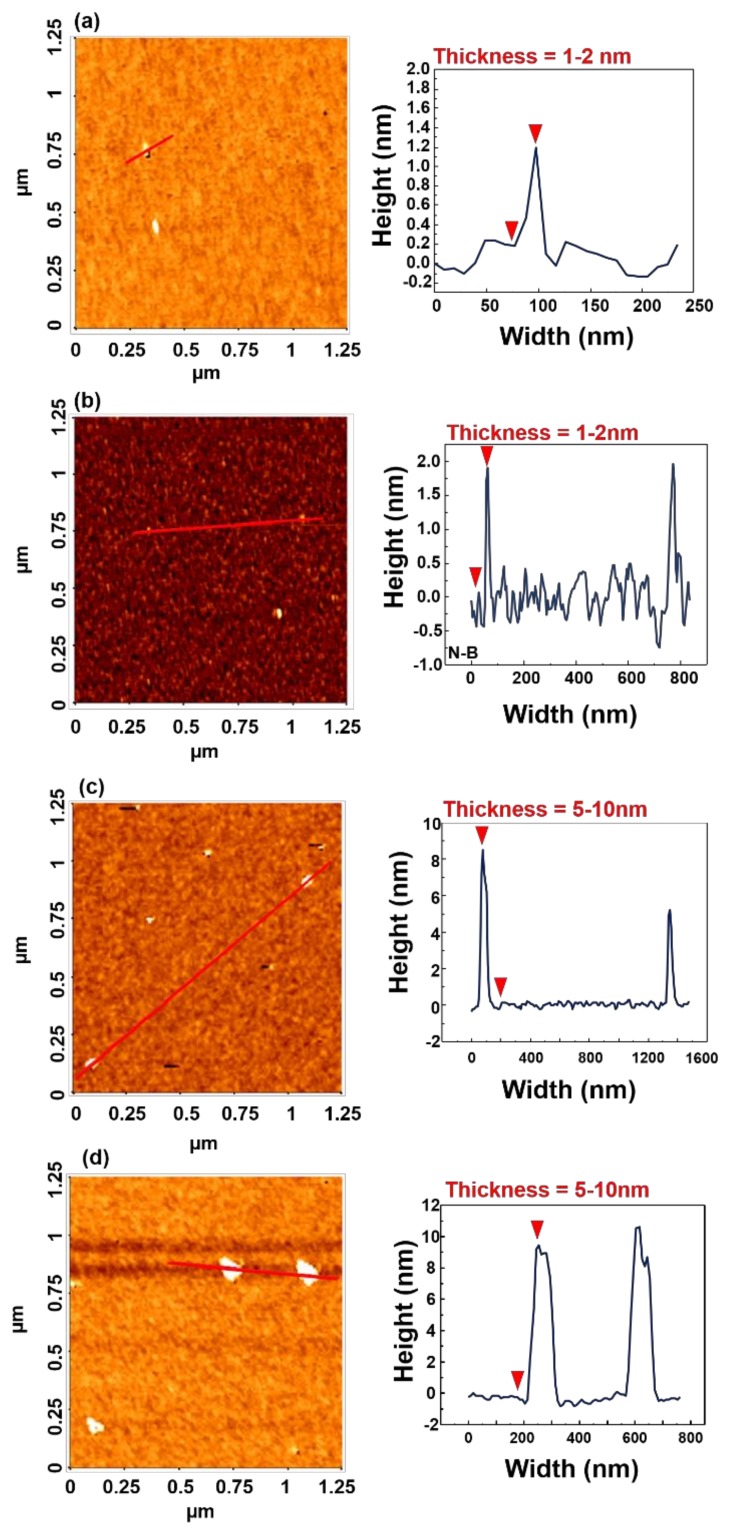
AFM images of (**a**) N-S, (**b**) N-B, (**c**) M-S, and (**d**) M-B. The width and height indicate the value of the red line in the left image.

Figure 4 indicates the thicknesses of nanosheets and multilayers, measured by AFM. The MoS_2_ nanoparticles dispersed in DI water were deposited on a silicon substrate, without spin-coating, for AFM measurement. N-S was shown to have a thickness of 1 nm, which roughly corresponds to a single layer [39]. The value of N-B was measured to be 2 nm, which indicated that N-B was bilayered and that the nanosheets were 1–2 layers thick. On the other hand, the thicknesses of M-S and M-B were between 5–10 nm, which would require more than 5 layers, at least, and these results indicated that nanosheets and multilayers could be clearly differentiated, based on relative thickness. 

**Figure 5:**
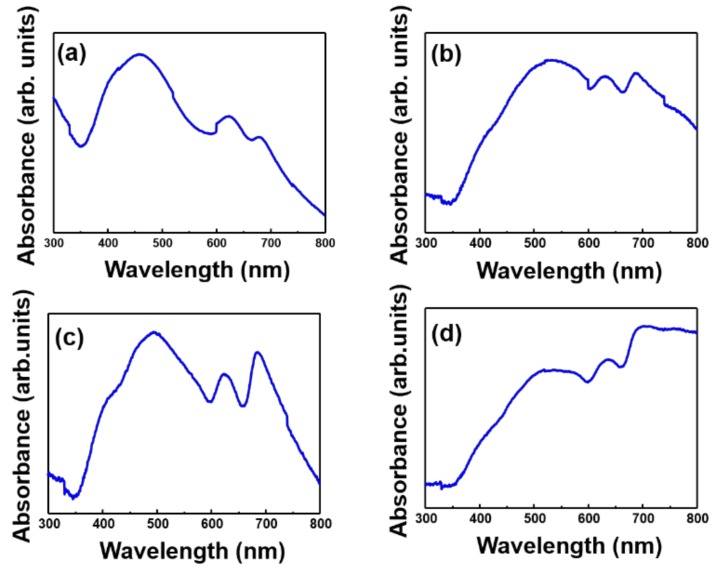
UV-vis spectra of (**a**) N-S, (**b**) N-B, (**c**) M-S, and (**d**) M-B. The difference in peak points for each graph is related to the change in the thickness of MoS_2_.

Figure 5 shows the UV-vis spectra of MoS_2_ of different sizes and thicknesses. N-S, which was mostly composed of a single layer, showed a peak at 622 nm and 678 nm. For N-B, the peak point shifted to 630.5 nm and 686.5 nm, due to the mixture of single and bilayers. When compared to the difference in the thickness of N-S and N-B in the AFM data, the imagery in Figure 5 is convincing. In previous research, for a single layer of the 2H phase, peaks appeared at 617 nm and 670 nm [40]. Since M-S is much thicker than a single layer, there is a big difference in peak point, while the peak point of M-B was seen to be similar to that for MoS_2_ raw material. Overall, the UV data showed results which were consistent with the results from the FE-SEM, DLS, and AFM analyses, as shown above.

### 3.2. Rheological Properties of WBM

**Figure 6:**
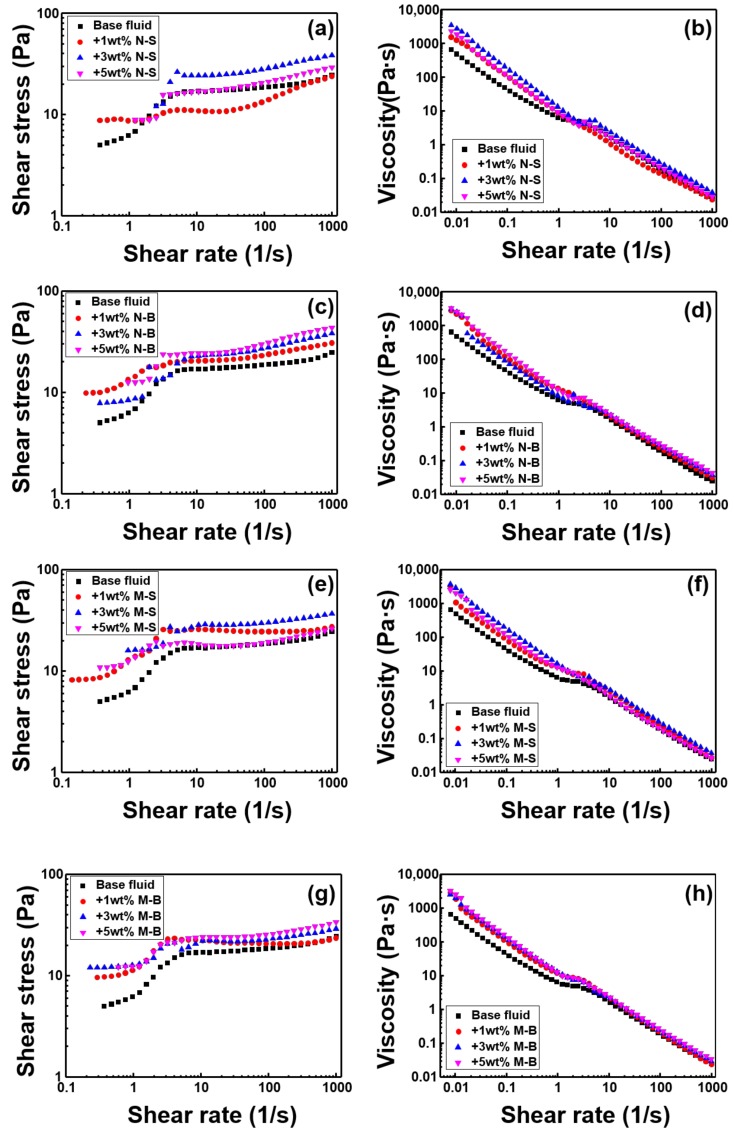
The shear stress and viscosity of WBM are classified according to the MoS_2_ particle size and compared with the particle concentration. (**a**) and (**b**) N-S, (**c**) and (**d**) N-B, (**e**) and (**f**) M-S, and (**g**) and (**h**) M-B.

Figure 6 shows the rheological properties. When the shear rate was very small, it showed positive shear stress, rather than 0, which indicated that all kinds of drilling mud have yield stress. MoS_2_ type N-S (Figure 3a) did not show a linear increase as the concentration increased to 1, 3, 5 wt %. Even the N-S 1 wt % graph shows that after passing through the intermediate peak point (shear rate = 1–10), the viscosity and shear stress were lower than those of the base fluid. The shear stress data shows that the slope of the increased shear stress, according to the shear rate, was less than 1. In the case of Newtonian fluids, considering the slope of 1, the drilling mud with N-S added was very strong, in terms of shear thinning material. Drilling mud with N-B added is also considered strong shear thinning material, as its shear rate was less than 1. In addition, the viscosity and shear stress of the drilling mud with N-B increased, in comparison with the base fluid, and there was a linear relationship between the concentration of N-B and the increase in shear stress, after a certain shear rate interval (1–10). When M-S was added, all of the rheological properties increased in comparison with the base fluid, although as the concentration increased, neither the shear stress, nor viscosity, nor even the yield stress, increased linearly. In order to obtain the correlation between the MoS_2_ addition concentration and increased shear stress, the N-S and M-S graphs were studied, and it could be confirmed that the diameter of MoS_2_ needed to be larger than a certain size. M-B also appeared to exhibit shear thinning, similarly to the other three MoS_2_ samples. In general, drilling mud shear stress and viscosity were enhanced linearly, with increasing concentration, although not at all points of the shear rate. When all MoS_2_ samples were added to the base fluid, viscosity and shear stress were increased at a lower shear rate than that of base fluid. It was also confirmed that MoS_2_ is a particle that strongly strengthened shear thinning.

**Figure 7:**
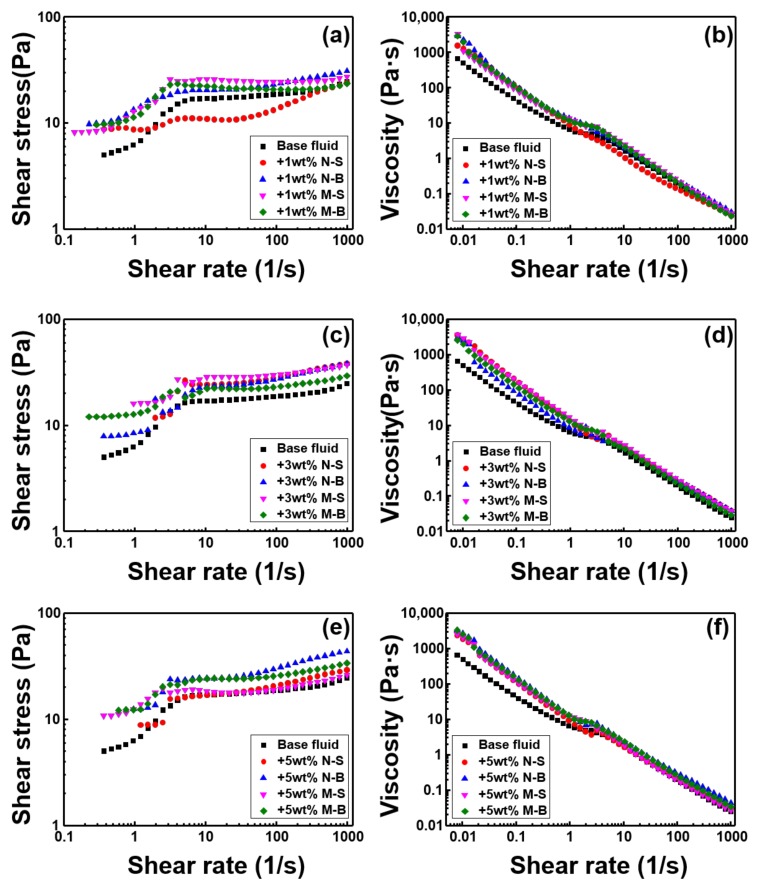
The shear stress and viscosity of WBM are compared according to the particle size at the same concentration of MoS_2_. (**a**) and (**b**) 1 wt %, (**c**) and (**d**) 3 wt %, (**e**) and (**f**) 5 wt %.

Figure 7 shows the rheological properties of MoS_2_ according to size and thickness at the same concentration. From the viewpoint of the viscous additive, the viscosity and shear stress were clearly increased when 1 wt % of MoS_2_ was added, in contrast to the base fluid, except for the N-S sample. However, Figure 7b shows N-S to be the most definitive shear thinning sample. When 3 wt % of MoS_2_ was added, M-S was the best performing sample, in terms of viscosity and shear stress, with all samples exhibiting shear thinning properties, even though there was a difference in degree. When 5 wt % MoS_2_ was added, N-B showed the most increase in viscosity and shear stress, in comparison with the base fluid. In the case of M-S and M-B, shear thinning could be confirmed, even though the surface area per unit mass of MoS_2_ is less than that of N-S or N-B, due to their thickness. It can be seen that the shear thinning property of MoS_2_ can be attributed to not only its two-dimensional structure but also to its low coefficient of friction.

### 3.3. Thermal Conductivity of WBM

**Figure 8:**
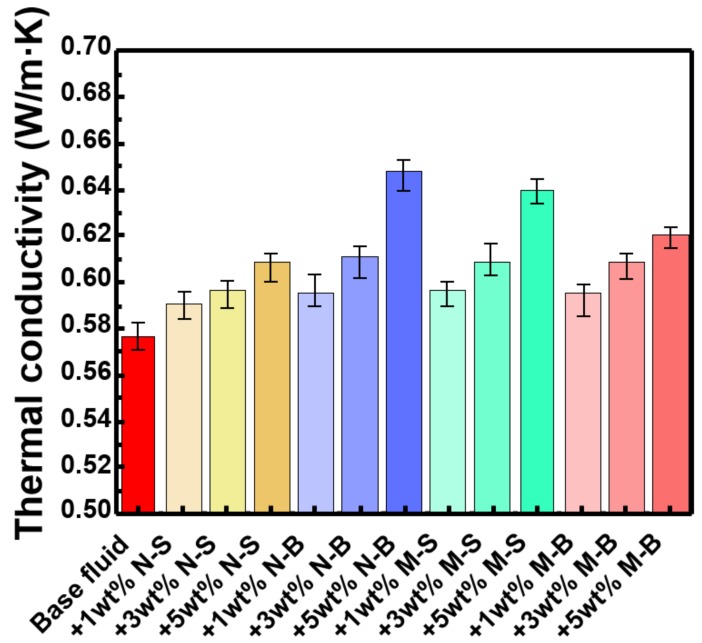
The thermal conductivity for each WBM. Each sample was measured three times and the average value was shown. As the concentration of MoS_2_ nanoparticle increases, the thermal conductivity increases linearly. Detailed data and the rate of increase can be found in Table S2.

Figure 8 shows the thermal conductivities of the base fluid, and of drilling fluid with MoS_2_ added. All drilling muds were enhanced linearly with increasing MoS_2_ concentration because the MoS_2_ thermal conductivity is much higher than that of the base fluid. The level of enhancement varied depending on the size and thickness of the MoS_2_ added, as MoS_2_ nanosheets have been shown to have lower thermal conductivity than MoS_2_ multilayers, plus, thermal conductivity varies with MoS_2_ particle diameter. It has been confirmed experimentally that the N-S sample has less increase in thermal conductivity with increasing concentration than other three samples. (N-B, M-S, M-B), when the three factors above were combined. It is noteworthy that even though N-B is a nanosheet, it showed the highest thermal conductivity increase rate, and it has been shown experimentally that the diameter of the MoS_2_ sample was also an important factor in determining thermal conductivity. The reason why the thermal conductivity of the M-S form was higher than that of the M-B form seems to be that surface area is important. In the case of a single layer, there was no difference in the surface area of MoS_2_, because it is a two-dimensional structure, however, since the multilayer structure has a three-dimensional structure, the M-B sample surface area must be smaller than that of M-S, while M-S exhibited higher values compared with M-B, for the same reason. Overall, N-B showed the highest degree of thermal conductivity increase, because while MoS_2_ nanosheets showed low thermal conductivity generally, the larger diameter and wider surface area of the 2D product, in comparison with the multilayer samples, gave rise to this result. The MoS_2_ M-S sample showed the second highest value, which was attributed to its comparative surface area and thickness. For the N-S sample, thickness and diameter are all of the low thermal conductivity forms, so that, even though it had a wide surface area, its increased thermal conductivity was comparatively low.

### 3.4. Optimization of MoS_2_ for WBM Additive

**Figure 9:**
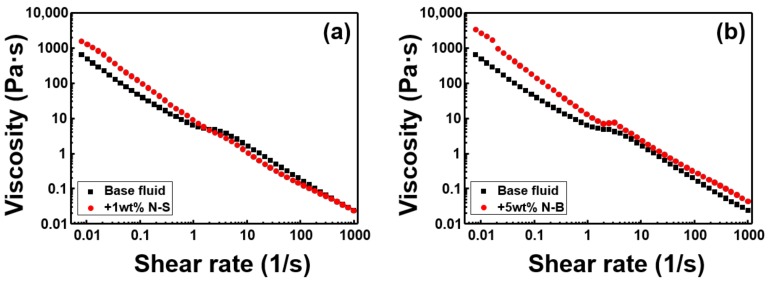
Shows the viscosity according to the shear rate of WBM. (**a**) WBM with 1 wt % N-S added to the base fluid showing the most obvious example of shear thinning, (**b**) WBM with 5 wt % N-B added, which is the case with the highest increase in viscosity and thermal conductivity.

Figure 9 is a rheological graph showing shear thinning and the highest thermal conductivity. In Figure 9a, the viscosity is higher than the base fluid, when the flow velocity is slower, and the viscosity becomes less as the flow velocity increases, in comparison to the base fluid. Figure 9a shows that even when the increase in thermal conductivity was minimal, the effect of shear thinning was quite clear. MoS_2_ serves as a viscous additive, but the relative drilling mud viscosity increase is often not proportional to the amount of MoS_2_ added. The most obvious increase in viscosity was when 5 wt % N-B was added to the mud, which also gave the best result for increased thermal conductivity, showing that it was suitable as a multifunctional mud additive, capable of simultaneously increasing drilling mud thermal conductivity and viscosity.

## 4. Conclusions

We synthesized bulk MoS_2_ nanoparticles by Li-intercalation and sonication. Nanosheets 1–2 nm thick were fabricated by the Li-intercalation method and were classified into two groups, small, with diameters between 100–400 nm, and large, with diameters between 300–600 nm, by applying different sonication power and centrifuge speed. Multilayers 5–10 nm thick were fabricated, by MoS_2_ raw material ultrasonication, in DI water. The multilayered forms were also divided into two groups, having small (100–400 nm), and large (400–650 nm) diameters, by adjusting sonication power and time. The four fabricated samples were then added to base fluid at 1, 3, and 5 wt %, depending on the concentration, and their resultant rheological and thermal conductivity properties were measured. MoS_2_ is a substance that generally increases shear stress and viscosity, but when the MoS_2_ diameter was small, the extent of increase was not linearly proportional to the concentration. Results also indicated that shear thinning properties, one of the important aspects of drilling mud, were related to the structure and low coefficient of friction of MoS_2_. MoS_2_ exhibited different thermal conductivity according to the thickness and diameter of the form in question, and overall, these properties were shown to be the most important factors of the MoS_2_ sample under consideration. As a result, we confirmed the optimal size and thickness of MoS_2_ nanosheets used as an additive in drilling mud and confirmed experimentally the effect of the nanosheets in shear thinning, viscosity increase, and increased thermal conductivity.

## Supplementary Materials

The following are available online at http://www.mdpi.com/2073-4360/11/2/321/s1, Figure S1: SEM images of bentonite, Table S1: WBM compositions, Table S2: WBM’s thermal conductivity.
