# Supplementary material for: Influence of MoS2 Nanosheet Size on Performance of Drilling Mud
Source: Polymers (Basel). 2019 Feb 13;11(2):321. doi: 10.3390/polym11020321 (PMC6419213; doi:10.3390/polym11020321)
Supplement: Supplementary file 1 [file polymers-11-00321-s001.pdf]

Supplementary Materials

Influence of MoS_2 nanosheet size on performance of drilling mud

Sung Hyun Hong [1], Hae Jin Jo [2], Min-Ju Choi [3], Ho Won Jang [3], Young Ju Kim [4,*], Wook Ryol Hwang [2,*] and Soo Young Kim [1,*]

[1] School of Chemical Engineering and Materials Science, Chung-Ang University, 84 Heukseok-ro, Dongjak-gu, Seoul 06974, Republic of Korea; hongthomas91@cau.ac.kr; sooyoungkim@cau.ac.kr

[2] School of Mechanical Engineering, Research Center for Aircraft Parts Technology (ReCAPT), Gyeongsang National University, Jinju, 52828, Republic of Korea; haejinjo@gnu.ac.kr; wrhwang@gnu.ac.kr

[3] Research Institute of Advanced Materials, Department of Materials Science and Engineering, Seoul National University, Seoul 08826, Republic of Korea; choiminju@snu.ac.kr; hwjang@snu.ac.kr

[4] Korea Institute of Geoscience and Mineral Resources, 905 Yeongilman-daero, Heunghae-eup, Buk-gu, Pohang-si, Gyeongsangbuk-do 37559, Republic of Korea; kyjp7272@kigam.re.kr

[*] Correspondence: Young Ju Kim: kyjp7272@kigam.co.kr; Tel.: +82-54-245-3780, Wook Ryol Hwang: wrhwang@gnu.ac.kr; Tel.: +82-55-772-1628, Soo Young Kim: sooyoungkim@cau.ac.kr; Tel.: +82-2-820-5875:

Received: date; Accepted: date; Published: date

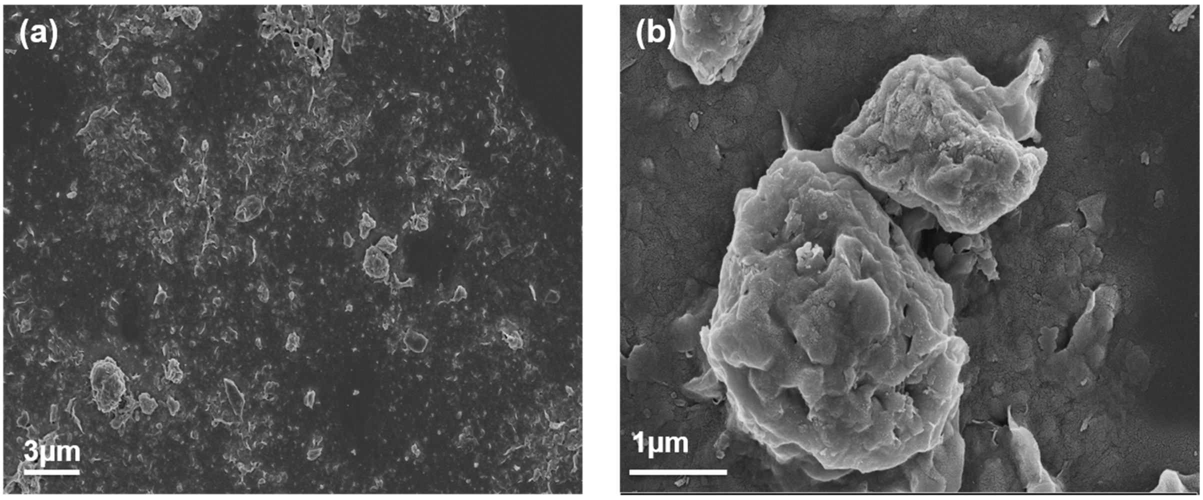

Figure S1. FE-SEM image of bentonite. (a) The particle size of bentonite is about 1 to 3 μm. (b) The particle shape appears to have a rough spherical surface.

Table S1. Table of WBM compositions. MoS_2 was divided into 4 kinds of N-S, N-B, M-S and M-B and concentrations of 1, 3 and 5wt% were added to the base fluid.

		MoS_2		
**WBM**	**Bentonite**	**Thickness**	**Diameter**	**Concentration**
**Base fluid**	5wt%	-	-	-
**+1wt% N-S**	5wt%	1-2 nm	100-400 nm	1wt%
**+3wt% N-S**	5wt%	1-2 nm	100-400 nm	3wt%
**+5wt% N-S**	5wt%	1-2 nm	100-400 nm	5wt%
**+1wt% N-B**	5wt%	1-2 nm	300-600 nm	1wt%
**+3wt% N-B**	5wt%	1-2 nm	300-600 nm	3wt%
**+5wt% N-B**	5wt%	1-2 nm	300-600 nm	5wt%
**+1wt% M-S**	5wt%	5-10 nm	100-400 nm	1wt%
**+3wt% M-S**	5wt%	5-10 nm	100-400 nm	3wt%
**+5wt% M-S**	5wt%	5-10 nm	100-400 nm	5wt%
**+1wt% M-B**	5wt%	5-10 nm	400-650 nm	1wt%
**+3wt% M-B**	5wt%	5-10 nm	400-650 nm	3wt%
**+5wt% M-B**	5wt%	5-10 nm	400-650 nm	5wt%

Table S2. The table of WBM's thermal conductivity. WBM with 5wt% nanosheets and big diameter MoS2 showed the highest thermal conductivity.

WBM	Thermal conductivity (W/m·K)	Rate of increase (%)
**Base fluid**	0.577	-
**+1wt% N-S**	0.591	2.4
**+3wt% N-S**	0.597	3.5
**+5wt% N-S**	0.609	5.5
**+1wt% N-B**	0.596	3.3
**+3wt% N-B**	0.611	5.9
**+5wt% N-B**	0.648	12.3
**+1wt% M-S**	0.597	3.5
**+3wt% M-S**	0.609	5.5
**+5wt% M-S**	0.639	10.7
**+1wt% M-B**	0.595	3.1
**+3wt% M-B**	0.609	5.5
**+5wt% M-B**	0.620	7.5
